# Undernourished and Undertreated: The Role of Nutritional Care in Geriatric Hospital Outcomes

**DOI:** 10.3390/nu17183021

**Published:** 2025-09-22

**Authors:** Paolo Orlandoni, Nikolina Jukic Peladic, Mirko Di Rosa, Claudia Venturini, Fabrizia Lattanzio

**Affiliations:** 1 Clinical Nutrition Unit, National Institute of Health and Science on Aging, IRCCS INRCA Ancona, Via Della Montagnola 81, 60127 Ancona, Italy; c.venturini@inrca.it; 2 Vivisol Srl., Clinical Nutrition Unit, National Institute of Health and Science on Aging, IRCCS INRCA Acona, Via Della Montagnola 81, 60127 Ancona, Italy; n.jukicpeladic@inrca.it; 3 Unit of Geriatric Pharmacoepidemiology and Biostatistics, National Institute of Health and Science on Aging, IRCCS INRCA Ancona, Via Santa Margherita 5, 60124 Ancona, Italy; m.dirosa@inrca.it; 4 Scientific Direction, IRCCS INRCA Ancona, Via Santa Margherita 5, 60124 Ancona, Italy; f.lattanzio@inrca.it

**Keywords:** malnutrition, in-hospital geriatrics patients, medical nutrition therapy, in-hospital mortality, length of hospital stay, GNRI

## Abstract

Background: Malnutrition is highly prevalent in hospitalized older adults and is associated with adverse clinical outcomes. However, the extent to which nutritional care is systematically implemented and its relationship with outcomes remains unclear. Methods: We conducted a retrospective analysis of 4963 patients aged ≥65 years who were admitted to the National Institute for Health and Scientific Research for the Elderly (IRCCS INRCA, Ancona, Italy) between 2012 and 2020 and received at least one nutritional consultation. We examined associations between timing and type of nutritional interventions, the Geriatric Nutritional Risk Index (GNRI), and clinical outcomes, including in-hospital mortality and length of hospital stay (LOS). We also analyzed the prevalence of different complications during Medical Nutritional Therapy (MNT). Results: Only 11% of hospitalized patients received a nutritional consultation. Among them, in-hospital mortality was 35.8%. The Geriatric Nutritional Risk Index was a significant predictor of mortality (HR 1.89; 95% CI: 1.55–2.31; *p* < 0.001) and inversely associated with LOS, reflecting a frail subset of patients with complex clinical conditions. The timing of nutritional consultation showed no significant association with mortality or LOS, possibly due to relatively prompt intervention (median 4 days). Enteral (EN) and parenteral nutrition (PN) were more frequently prescribed to non-survivors. Medical nutrition therapy-related complications were also more common in non-survivors (22.3% vs. 14%, *p* < 0.001). Artificial nutrition (AN) was not associated with increased mortality, but it was associated with prolonged hospital stays both in survivors and in non-survivors. Conclusions: Our findings highlight the limited use of nutritional consultations in hospitalized elderly patients despite the high prevalence of malnutrition and its prognostic relevance. The GNRI is a valuable tool for early risk stratification and clinical decision-making. Systematic screening and timely nutritional intervention, integrated with broader therapeutic goals, may improve care and optimize outcomes even in this vulnerable population.

## 1. Introduction

The progressive aging of the global population is a continuing trend that shows no signs of reversing [1]. Instead, it presents modern society with numerous challenges, chief among them the management of health-related issues and the resulting hospitalizations. According to the Global Burden of Disease study, approximately 23% of the total disease burden in 2010 was attributable to individuals aged 60 years and older [2]. In Italy, where expenditures for acute care hospitalizations account for nearly half of total healthcare spending, over 50% of these costs are attributable to individuals aged 65 years and older [3].

Malnutrition—a condition resulting from an imbalance between nutrient intake and individual nutritional requirements—is both a cause of hospitalization in older adults and a factor contributing to prolonged hospital stays for underlying diseases. Advanced age, chronic comorbidities, polypharmacy, and inflammation are key contributors that increase energy expenditure and impair nutrient absorption and assimilation, ultimately leading to undernutrition [4,5,6].

Numerous social and psychological factors—including loneliness, social isolation, depression, and neurological impairments—also negatively influence dietary intake and contribute to malnutrition in this population [7]. In addition, during hospitalization, food intake is often further reduced due to pre- and post-operative fasting, poor palatability of hospital meals, concerns about health status, and uncertainty regarding clinical outcomes [8,9,10,11,12].

It is estimated that between 40% and 80% of older adults are already malnourished upon hospital admission, while 10% to 65% develop hospital-acquired malnutrition, typically occurring within the first 10 days of hospitalization, accounting for up to 70% of cases [13,14,15]. Despite this, nutritional care remains a low priority in many hospital settings. It is estimated that in 60% to 70% of cases, patients’ nutritional status is neither assessed nor treated during their hospital stay [16].

Undernutrition adversely affects the recovery of functional status, increases the risk of postoperative complications, prolongs the length of hospital stay (LOS) by up to 70%, and raises the risk of mortality and early readmission following discharge [17,18,19]. This creates a vicious cycle that significantly burdens both the patient and the healthcare system.

In this retrospective study, we analyzed data from 4963 older patients hospitalized at the geriatric hospital IRCCS INRCA in Ancona (Italy) between 2012 and 2020, all of whom received a nutritional care plan. The primary objectives of this study were to:

(a) describe how nutritional issues are currently managed in a geriatric hospital setting;

(b) assess the in-hospital mortality rate among malnourished geriatric patients;

(c) evaluate the effectiveness of the Geriatric Nutritional Risk Index (GNRI) in predicting in-hospital mortality and length of stay;

(d) estimate the impact of early nutritional intervention and Medical Nutrition Therapy (MNT)—particularly Enteral Nutrition (EN) and Parenteral Nutrition (PN)—on reducing in-hospital mortality and shortening the length of hospital stay [20].

## 2. Materials and Methods

This retrospective observational study included patients aged over 65 years who were hospitalized at IRCCS INRCA in Ancona (Italy) between 2012 and 2020, and who participated in the Reportage study (Trial registration number: NCT01397682; registered on 19 July 2011). The National Institute for Health and Scientific Research for the Elderly has 169 available beds in Ancona. The median Length of Hospital Stay in last four years was 8 days. As part of the research activity, in 2011 was started the Reportage study. The detailed methodology of the study has been described elsewhere [21]. The Reportage study was a prospective observational study involving older adults admitted to INRCA research hospitals in Italy. For our analysis, only patients hospitalized in Ancona were included. Eligible participants were those who had received at least one nutritional consultation from the Clinical Nutrition Unit—comprising physician nutrition specialists and dietitians—and had been prescribed nutritional therapy. A total of 4963 patients met the inclusion criteria and were analyzed.

The data were retrieved from the Reportage database and from the Clinical Nutrition Unit database.

Baseline characteristics were collected on the first day of hospital admission by ward staff and included date of hospitalization, sex, age, comorbidities, and primary reason for admission. Those data were retrieved from Reportage database. The Clinical Nutrition Unit conducted nutritional screening and assessments upon request from the ward for patients who were unable or refused to eat. For the nutritional screening, Malnutrition Universal Screening Tool (MUST) was used. During these assessments, the following data were collected and deposited in a dedicated database: body weight, height, presence of pressure ulcers, and laboratory test results, including serum albumin, prealbumin, total protein, and C-reactive protein (CRP). Body Mass Index (BMI) was calculated for all patients using the standard formula: weight in kilograms divided by height in meters squared (kg/m^2^) [22].

The Geriatric Nutritional Risk Index (GNRI), a validated tool for predicting morbidity and mortality risk in hospitalized elderly patients, was also calculated [23]. The GNRI was determined using the following formula:

GNRI = (1.489 × serum albumin [g/L]) + (41.7 × present weight [kg]/ideal weight [kg])

Ideal body weight was estimated using the Lorentz equations, as indicated by Bouillanne et al. [23]:

- For men: Ideal weight (kg) = 0.75 × height (cm) − 62.5
- For women: Ideal weight (kg) = 0.60 × height (cm) − 40.

Additional nutrition-related data collected during the nutritional assessment included: pre-assessment diet (i.e., food or nutrients provided via the oral route) and/or nutritional therapy (i.e., a medical treatment including oral, enteral, and/or parenteral Nutrition), diet or MNT prescribed after the consultation from the Clinical Nutrition Unit (i.e., screening and assessments performed by the staff of Clinical Nutrition Unit), number of nutritional consultations, date of nutritional intervention initiation, and any nutrition-related complications. Nutrition-related complications were defined by observing the changes in clinical conditions and biochemical values, after the initiation of nutritional therapy and after other possible causes, such as infections, underlying diseases, drug therapies, and others were excluded.

### Statistical Analyses

Medians with interquartile range were used to describe continuous variables with non-normal distribution (assessed with the Shapiro–Wilk test); absolute frequencies and percentages were used for categorical variables. The Chi-square test and the Mann–Whitney U test were performed to compare variables between groups (survived vs. deceased patients), as appropriate. The association of GNRI, Artificial Nutrition (AN) during hospitalization and, separately, EN or PN during hospitalization with in-hospital mortality was then assessed by using Kaplan- Meier survival curves and statistical significance was assessed via Log-rank test equality of survivor functions. Two multivariate Cox regression models for each independent variable were estimated: Model 1 was s adjusted for age, gender and main comorbidities (i.e., Neurological disease, Lung disease, Kidney disease, Cancer, Infection, Malnutrition, Cardiovascular disease, and Diabetes mellitus); Model 2 was adjusted for age, gender and pluripathology (i.e., at least one of the aforementioned comorbidities). The same models were then used to estimate multivariate linear regressions to find potential determinants of prolonged length of hospital stay. All the analyses were performed with Stata MP 19.0 (StataCorp LP, College Station, TX, USA). Differences were considered statistically significant with *p* values less than 0.05 (two-sided).

## 3. Results

### 3.1. Baseline Characteristics of Patients

A total of 4963 patients hospitalized across various wards of the geriatric hospital IRCCS INRCA in Ancona between 2012 and 2020, and who received at least one consultation from the Clinical Nutrition Unit following the request of medical doctors from those wards, were included in the study. During the study period, 11.44% of all patients admitted to INRCA Ancona received nutritional consultations.

Baseline characteristics of the study population, stratified into ‘survivor’ (i.e., patients discharged after the hospitalization) and ‘non-survivor’ (i.e., patients who died during hospitalization) groups, are presented in Table 1. The overall all-cause in-hospital mortality rate was 35.8%.

The median age of the study population was 88 years (interquartile range [IQR]: 84–92), with females comprising 61.1% and males 38.9%. Statistically significant differences were observed between survivors and non-survivors in most baseline clinical characteristics. Relatively to comorbidities, lung and kidney diseases, as well as type 2 diabetes mellitus, were more common among non-survivors, while neurological diseases were the most prevalent comorbidity (overall 61.4%) and the leading cause of hospitalization (overall 24.8%) in both groups.

Body Mass Index was significantly lower in the non-survivor group (median 21.4 kg/m^2^ [IQR 18.8–24.2]) compared to survivors (median 22.0 kg/m^2^ [IQR 19.4–24.7]; *p* < 0.001). With the screening that was performed using MUST, a high risk of malnutrition was found for all subjects (score 2 or more). According to the Geriatric Nutritional Risk Index, non-survivors had a significantly higher nutritional risk at baseline (*p* < 0.001), with 56% classified as high risk, compared to 46.5% among survivors.

Before the nutritional assessment, 55.2% of non-survivors were receiving only hydration, in contrast to 41.8% of survivors. Additionally, only 27.6% of non-survivors were consuming a regular oral diet, compared to 37.8% of survivors. The median length of hospital stay was 10 days (IQR: 5–22), with no statistically significant difference between the two groups (*p* = 0.197).

### 3.2. Features of Nutrition Treatment

The median time from hospital admission to nutritional assessment by the Clinical Nutrition Unit was 4 days (IQR 2–7), with a statistically significant difference between survivors and non-survivors (4 days [IQR 2–7] vs. 4 days [IQR 2–8], respectively; *p* = 0.007) (See Table 2).

Most patients received only one nutritional consultation during hospitalization. However, non-survivors underwent a significantly higher number of consultations compared to survivors (22.2% of them had two consultations, 12.7% had three or more; *p* < 0.001). All nutritional plans were significantly modified following the initial consultation. The proportion of patients maintained on a free diet or receiving only hydration decreased significantly. Oral supplements were only 6.5% in survivors and even less (2.2%) in no-survivors, while AN, EN and PN, became highly prevalent—56.7% in survivors and 65.4% in non-survivors. Parenteral nutrition was prescribed after the first consultation to 43.3% of non-survivors and 34.8% of survivors, whereas enteral nutrition was prescribed to approximately 20% of patients in both groups. Hydration alone was more common among non-survivors (21% vs. 11.8% in survivors), while the use of texture-modified oral diets for dysphagia increased significantly among survivors (16.7% vs. 7.9% in non-survivors). Among 991 patients (30.1% of the total) who received at least a second nutritional consultation, nutritional therapy was further modified in 40.6% of cases (*n* = 597). Overall, PN was administered throughout the entire hospitalization period to 56.6% of non-survivors compared to 42.2% of survivors (*p* < 0.001). No significant difference was observed in the prevalence of EN between the two groups (29.5% in survivors vs. 29.7% in non-survivors; *p* = 0.849).

**Figure 1:**
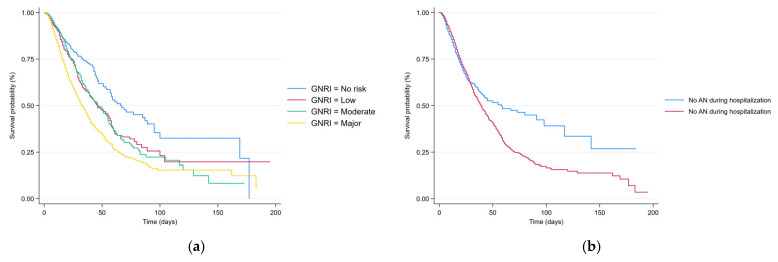

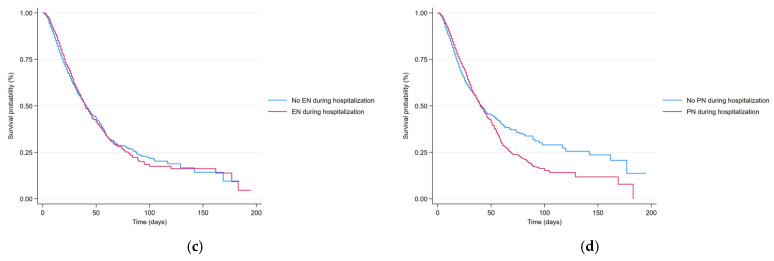
Kaplan–Meier survival curves. (**a**) In-hospital mortality for different GNRI (*p* < 0.001). (**b**) In-hospital mortality AN during hospitalization vs. no AN (*p* = 0.034). (**c**) In-hospital mortality EN during hospitalization vs. no EN (*p* = 0.299). (**d**) In-hospital mortality PN during hospitalization vs. no PN (*p* = 0.593).

Kaplan–Meier curves (Figure 1) were used to analyze the in-hospital mortality and how it is influenced by GNRI (a), and nutritional therapies (AN (b), EN (c), PN (d)) administered throughout the entire hospitalization period. Statistical significance emerged only for GNRI (especially comparing the No risk vs. Major risk categories) and for AN during hospitalization.

Nutrition-related complications were recorded in 791 patients (16.9%), with a significantly higher incidence in non-survivors compared to survivors (22.3% vs. 14.0%; *p* < 0.001). The most notable differences were observed in electrolyte imbalances and glycemic alterations, which occurred more frequently among non-survivors (9.7% vs. 4.9%, *p* < 0.001, and 1.6% vs. 0.7%, *p* = 0.003, respectively).

To evaluate the association between clinical variables—including GNRI, time from hospital admission to nutritional assessment, AN, PN—and in-hospital mortality, multivariate analysis was conducted using Cox proportional hazards models (see Table 3).

As shown in Table 3, both Model 1 (adjusted for age, sex, and individual comorbidities) and Model 2 (adjusted for age, sex, and multimorbidity) demonstrated a significant association between GNRI categories and all-cause in-hospital mortality. Compared to patients with no nutritional risk, hazard ratios (HR) in Model 1 were 1.28 (95% CI: 1.02–1.61; *p* = 0.033) for low risk, 1.25 (95% CI: 1.01–1.55; *p* = 0.038) for moderate risk, and 1.89 (95% CI: 1.55–2.31; *p* < 0.001) for major risk. Similarly, Model 2 showed HRs of 1.31 (95% CI: 1.05–1.65; *p* = 0.019), 1.28 (95% CI: 1.04–1.58; *p* = 0.023), and 1.96 (95% CI: 1.61–2.39; *p* < 0.001), respectively.

The association between AN during hospitalization and all-cause in-hospital mortality was significant only in Model 1 (HR = 1.14; 95% CI: 1.01–1.28; *p* = 0.030). No statistically significant associations were found between PN administration or the interval between hospital admission and nutritional consultation and in-hospital mortality in either model.

To examine the association between GNRI, the timeliness of nutritional intervention, different nutritional therapies, and length of hospital stay (LOS), linear regression analyses were performed (see Table 4).

GNRI was negatively associated with LOS, indicating that patients with higher nutritional risk—who are more susceptible to mortality and complications—tended to have shorter hospital stays, possible due to earlier in-hospital mortality (see Table 4). The promptness of nutritional intervention showed no significant association with LOS. Conversely, both PN and EN administered during hospitalization were associated with prolonged LOS in both models. Specifically, EN was associated with an increase in LOS of 1.88 days (*p* = 0.010) in Model 1 and 1.98 days (*p* = 0.005) in Model 2, while PN was associated with a more pronounced increase of 6.84 days (*p* < 0.001) in Model 1 and 6.58 days (*p* < 0.001) in Model 2.

Overall, the AN during hospitalization was associated with a prolonged LOS in both Model 1 and 2 (5.87 days (*p* < 0.00) vs. 5.67 days (*p* < 0.001)). The supporting information can be found in Tables S1 and S2.

## 4. Discussion

To evaluate the current management of nutritional care in a geriatric hospital setting and to investigate the associations between timing and type of nutritional interventions, the Geriatric Nutritional Risk Index and clinical outcomes such as in-hospital mortality and length of hospital stay, we retrospectively analyzed data from 4963 patients aged ≥65 years. These patients were admitted to IRCCS INRCA in Ancona, Italy, between 2012 and 2020 and received at least one nutritional consultation.

Our findings indicate that only slightly more than 11% of hospitalized patients received nutritional counseling. Among these, 35.8% died during hospitalization. The GNRI proved to be a reliable predictor of in-hospital mortality. However, the timing of nutritional consultation was not significantly associated with either mortality or LOS. Following nutritional consultation, a notable proportion of patients (76.9%) received AN; nevertheless, both EN and, in particular, parenteral nutrition PN, were more frequently prescribed in non-survivors. Enteral and parenteral nutrition were not independently associated with mortality but they were both associated with significantly prolonged hospital stays (e.g., PN increased LOS by 6.84 days, *p* < 0.001). Nutrition-related complications were significantly more frequent among non-survivors (22.3% vs. 14.0%, *p* < 0.001).

A key observation from this study is the remarkably low proportion of patients receiving nutritional counseling, far fewer than the number likely to benefit from such assessment and intervention. Nutritional consultations were typically requested from wards only for patients who were unable or unwilling to eat, while systematic nutritional screening was not routinely performed. This reflects the limited priority given to nutritional care in clinical practice. Comparable trends have been reported in the literature for other hospitals, underscoring a widespread under-recognition of the issue. Furthermore, the ongoing debate within the scientific community regarding the most appropriate screening tools for assessing malnutrition risk adds to the challenge of implementing standardized nutritional care. This occurs despite the availability of advanced technologies, including artificial intelligence-based tools, which offer greater accuracy than traditional screening methods and hold promise for improving the integration of nutritional management into routine clinical practice [24]. As shown in Table 1, although a statistically significant difference in BMI was found between survivors and non-survivors at baseline, the median values for both groups were within the ideal range (21.4 for non-survivors vs. 22.00 for survivors), while according to the screening test (MUST), all patients were at high risk of malnutrition. This data further confirms that BMI can only be used as a rough indicator of a subject’s nutritional status. In geriatric patients, its value is further reduced by the characteristics that distinguish this population (loss of muscle mass, changes in body composition, edema, and fluid retention). Furthermore, the BMI assessed at baseline does not take into account that most elderly patients hospitalized for acute conditions present hypercatabolism, i.e., inflammation or stress, and that a high number of subject will experience accelerated weight loss in the days following hospitalization due to pre- and post-operative fasting, poor satisfaction with hospital meals, or psychological reasons.

In-hospital mortality in our cohort was notably high (35.8%). It is important to emphasize that this figure reflects all-cause mortality, rather than deaths directly attributable to nutritional status. Moreover, the study population does not represent the totality of hospitalized geriatric patients, but specifically those who received a nutritional consultation—a subgroup that likely comprises a particularly vulnerable segment of an already frail population. For these reasons, direct comparison with mortality data from broader geriatric cohorts in other studies is not appropriate.

Consistent with previous research, the GNRI emerged as a robust predictor of in-hospital mortality in our study. It remained significantly associated with mortality in multivariate analyses (e.g., high-risk GNRI: HR 1.89; 95% CI: 1.55–2.31; *p* < 0.001). Higher nutritional risk, as indicated by the GNRI, was inversely associated with length of hospital stay, likely reflecting the earlier death of more severely compromised patients. These findings support the use of GNRI at admission as a valuable tool not only for mortality risk stratification but also for guiding early and individualized nutritional interventions.

The timing of nutritional intervention, specifically the interval between hospital admission and the first consultation by the nutritional team, was not significantly associated with either mortality or LOS in our analysis. However, existing evidence indicates that nutritional status tends to deteriorate within the first 10 days of hospitalization, and in our cohort, the median time to nutritional consultation was 4 days, so that the intervention was very rapid. Furthermore, although statistically significant differences in timing were observed between survivors and non-survivors, the overall timing of intervention can still be considered timely across both groups. This may partly explain the lack of association with clinical outcomes such as mortality or LOS, and suggests that further research is needed to determine the optimal window for nutritional assessment and intervention. Relative to the number of consultations, it was found that patients who did not survive hospitalization received more nutritional consultations. The conditions of critical patients indeed change quickly, requiring more adjustments to therapies and greater attention to potential complications.

At first glance, the prevalence of medical nutrition therapy (MNT), including oral nutritional supplements and AN, observed in our study appears markedly different than that reported in the existing literature—particularly with regard to a high prevalence of EN and PN [25,26]. However, this apparent discrepancy is attributable to the fact that these prevalence rates were calculated exclusively within the subset of hospitalized patients (approximately 11%) who received nutritional counseling. When the use of EN and PN is considered about the entire population of hospitalized geriatric patients during the study period, the resulting prevalence estimates align much more closely with those reported in previous studies [27,28].

Artificial nutrition, especially parenteral nutrition, was more frequently prescribed in non-survivors. Neither EN or PN were associated with in-hospital mortality, but they were both associated with significantly prolonged LOS in survivors and non-survivors (e.g., PN increased LOS by 6.84 days, *p* < 0.001). Nutrition-related complications were more frequent in non-survivors (22.3% vs. 14%, *p* < 0.001) and they also received a higher number of nutritional consultations during hospitalization. These observations may reflect the greater clinical complexity and severity of illness in this subgroup, which likely necessitated more intensive monitoring and support, but from this data a discussion could also arise suggesting that patients with poorer prognoses are often subjected to more resource-intensive hospitalizations, including a higher frequency of procedures and interventions. Data on LOS in older patients on PN are very rare so that is not possible to compare our results with results reported in other studies [29].

It is important however to underline that though our data on prolonged LOS could arise doubts about its economical sustainability, its use is clinically justified. In the first place, as the ESPEN guidelines on the ethical aspects of artificial nutrition and hydration emphasize, particularly in elderly patients, it is often not possible to immediately make a clear, evidence-based decision regarding the most appropriate therapeutic intervention [30]. In such cases, the principle in dubio pro vita (when in doubt, favor life) should be applied, guiding clinicians to continue supportive measures, including nutritional therapy, until a more definitive clinical judgment can be made. Second, the management of patients requiring invasive access devices such as midline catheters, central venous catheters (CVC), or port systems (CVP) inherently involves longer evaluation periods and greater clinical complexity. These interventions demand careful consideration, patient stabilization, and close monitoring to ensure safety and efficacy, particularly in a frail geriatric population [31]. Finally, the primary objective of nutritional therapy in hospitalized elderly patients is rarely purely nutritional. Rather, its main role is to prevent further deterioration of nutritional status, thereby preserving physiological reserves and enhancing the effectiveness of other therapeutic interventions aimed at the underlying or predominant medical condition [32]. In this context, nutritional support serves as a critical adjunct to overall clinical management rather than an isolated goal.

Although our study has several important strengths—such as a large sample size, a long observation period, and the use of validated screening tools—several limitations should be acknowledged. First, its retrospective design limits the ability to establish causal relationships between nutritional interventions and clinical outcomes. Second, the study population, which consisted of particularly old, frail patients, with multiple comorbidities, may have introduced confounding factors that influenced both the nutritional care provided and the outcomes observed. Comorbidities that characterize this population involve the use of a high number of drugs—polypharmacy—which, in turn, can have important implications for malnutrition. This variable was not included in the study due to the amount of missing data. Finally, as this study reflects the practices of a single center, some findings may not be directly generalizable to other healthcare settings.

## 5. Conclusions

Malnutrition management should be initiated at the onset of hospitalization, with systematic nutritional screening conducted across all hospital wards to prevent the underutilization of nutritional assessments and interventions. Such an approach may contribute to enhancing patients’ overall clinical condition. The Geriatric Nutritional Risk Index serves as a reliable tool for the early detection of individuals at nutritional risk and facilitates evidence-based clinical decision-making.

## Figures and Tables

**Table 1:** Baseline characteristics of patients who received at least one consultation from the Clinical Nutrition Unit (IRCCS INRCA 2012–2020); total number, survivor group and non-survivor group.

	Total	Survivor Group	Non-Survivor Group	*p*
	N = 4687 (100.0%)	N = 3008 (64.2%)	N = 1679 (35.8%)	
Gender *				<0.001
F	2865 (61.1%)	1921 (63.9%)	944 (56.2%)	
M	1822 (38.9%)	1087 (36.1%)	735 (43.8%)	
Age ^	88 (84–92)	87 (83–91)	89 (85–92)	<0.001
Pluripathology *	3690 (78.7%)	2327 (77.4%)	1363 (81.2%)	0.002
Pressure ulcers *	1957 (41.8%)	1186 (39.4%)	771 (45.9%)	<0.001
Laboratory parameters				
Albumin (mg/L) ^	3 (2.6–3.4)	3 (2.6–3.5)	2.9 (2.5–3.4)	<0.001
Prealbumin ^	11.8 (7.9–16.2)	12 (8.2–16.3)	11.25 (7.2–15.7)	0.023
Total proteins ^	5.9 (5.3–6.5)	5.9 (5.4–6.5)	5.9 (5.2–6.5)	0.289
CRP ^	5.56 (1.98–11.15)	5.16 (1.77–10.31)	6.34 (2.42–12.72)	<0.001
Diseases				
Other diseases *	1067 (22.8%)	701 (23.3%)	366 (21.8%)	0.238
Neurological disease *	2877 (61.4%)	1876 (62.4%)	1001 (59.6%)	0.064
Lung disease *	826 (17.6%)	488 (16.2%)	338 (20.1%)	0.001
Kidney disease *	1035 (22.1%)	566 (18.8%)	469 (27.9%)	<0.001
Gastrointestinal disease *	0 (0%)	0 (0%)	0 (0%)	-
Cancer *	573 (12.2%)	365 (12.1%)	208 (12.4%)	0.799
Infection *	29 (0.6%)	17 (0.6%)	12 (0.7%)	0.531
Malnutrition *	18 (0.4%)	14 (0.5%)	4 (0.2%)	0.228
Cardiovascular disease+	2400 (51.2%)	1493 (49.6%)	907 (54%)	0.004
Diabetes mellitus II *	1003 (21.4%)	638 (21.2%)	365 (21.7%)	0.672
Reason for hospitalization *				<0.001
Artificial Nutrition *	54 (1.2%)	47 (1.6%)	7 (0.4%)	
Aspiration pneumonia *	230 (4.9%)	146 (4.9%)	84 (5%)	
Cancer *	190 (4.1%)	125 (4.2%)	65 (3.9%)	
Cardiovascular disease *	343 (7.3%)	191 (6.3%)	152 (9.1%)	
Electrolytic alteration *	142 (3%)	95 (3.2%)	47 (2.8%)	
Gastrointestinal disease *	512 (10.9%)	384 (12.8%)	128 (7.6%)	
Infection *	493 (10.5%)	337 (11.2%)	156 (9.3%)	
Kidney disease *	137 (2.9%)	82 (2.7%)	55 (3.3%)	
Lung disease *	613 (13.1%)	351 (11.7%)	262 (15.6%)	
Malnutrition *	289 (6.2%)	203 (6.7%)	86 (5.1%)	
Metabolic alteration *	22 (0.5%)	13 (0.4%)	9 (0.5%)	
Neuropsychiatric disease *	1163 (24.8%)	710 (23.6%)	453 (27%)	
Other *	499 (10.6%)	324 (10.8%)	175 (10.4%)	
BMI ^	21.8 (19.1–24.6)	22 (19.4–24.7)	21.4 (18.8–24.2)	<0.001
GNRI ^	82.10 (74.07–90.38)	83.39 (75.14–90.84)	80.41 (71.78–89.35)	<0.001
GNRI Risk *	370 (7.9%)	258 (8.6%)	112 (6.7%)	<0.001
No risk *	645 (13.8%)	424 (14.1%)	221 (13.2%)	
Low *	1333 (28.4%)	927 (30.8%)	406 (24.2%)	
Moderate *	2339 (49.9%)	1399 (46.5%)	940 (56%)	
Major *				
Nutrition before the nutritional consultation *				
				<0.001
By mouth *	1601 (34.2%)	1138 (37.8%)	463 (27.6%)	
EN *	312 (6.7%)	230 (7.6%)	82 (4.9%)	
Hydration *	2182 (46.6%)	1256 (41.8%)	926 (55.2%)	
Liquid diet *	83 (1.8%)	62 (2.1%)	21 (1.3%)	
PN *	155 (3.3%)	99 (3.3%)	56 (3.3%)	
Missing *	354 (7.6%)	223 (7.4%)	131 (7.8%)	
Length of hospitalization ^	10 (5–22)	10 (5–21)	11 (5–24)	0.197

* Absolute frequencies (relative frequencies); ^ Median (IQR).

**Table 2:** The features of nutrition treatment in patients who received at least one consultation from the Clinical Nutrition Unit (IRCCS INRCA 2012–2020).

	Total	Survivor Group	Non-Survivor Group	*p*
	N = 4687 (100. 0%)	N = 3008 (64.2%)	N = 1679 (35.8%)	
Days from hospitalization to nutritional consultation ^	4 (2–7)	4 (2–7)	4 (2–8)	0.007
Nutrition therapy first visit *				<0.001
Diet for Dysphagia *	635 (13.5%)	503 (16.7%)	132 (7.9%)	
Diet for a specific pathology *	115 (2.5%)	98 (3.3%)	17 (1%)	
EN *	1030 (22%)	659 (21.9%)	371 (22.1%)	
Free diet *	116 (2.5%)	102 (3.4%)	14 (0.8%)	
Hydration *	709 (15.1%)	356 (11.8%)	353 (21%)	
Liquid diet *	26 (0.6%)	18 (0.6%)	8 (0.5%)	
Oral Supplements *	232 (4.9%)	195 (6.5%)	37 (2.2%)	
Other *	50 (1.1%)	30 (1%)	20 (1.2%)	
PN *	1774 (37.8%)	1047 (34.8%)	727 (43.3%)	
EN during hospitalization *	1385 (29.5%)	886 (29.5%)	499 (29.7%)	0.849
PN during hospitalization *	2221 (47.4%)	1270 (42.2%)	951 (56.6%)	<0.001
N. nutritional consultations *				<0.001
1	3217 (68.6%)	2124 (70.6%)	1093 (65.1%)	
2	991 (21.1%)	619 (20.6%)	372 (22.2%)	
3	479 (10.2%)	265 (8.8%)	214 (12.7%)	
Time between the nutritional consultation and the end of hospitalization ^	16 (10–29)	15 (10–28)	18 (10–31)	0.014
Complications				
Aspiration pneumonia *	18 (0.4%)	6 (0.2%)	12 (0.7%)	0.006
Electrolytic alteration *	311 (6.6%)	148 (4.9%)	163 (9.7%)	<0.001
Glycemic alteration *	48 (1%)	21 (0.7%)	27 (1.6%)	0.003
Diarrhea *	90 (1.9%)	50 (1.7%)	40 (2.4%)	0.085
Bronchial secretion *	20 (0.4%)	8 (0.3%)	12 (0.7%)	0.024
Dislocation VC and PN *	14 (0.3%)	10 (0.3%)	4 (0.2%)	0.571
NGT dislocation *	178 (3.8%)	117 (3.9%)	61 (3.6%)	0.660
Total complications *				<0.001
0	3893 (83.1%)	2588 (86%)	1305 (77.7%)	
1	628 (13.4%)	328 (10.9%)	300 (17.9%)	
2	144 (3.1%)	83 (2.8%)	61 (3.6%)	
3	21 (0.4%)	9 (0.3%)	12 (0.7%)	
4	1 (0%)	0 (0%)	1 (0.1%)	

* Absolute frequencies (relative frequencies); ^ Median (IQR).

**Table 3:** Cox proportional hazards model.

	MODEL 1		MODEL 2	
	HR (95% CI)	*p*	HR (95% CI)	*p*
GNRI, ref. No risk				
Low	1.28 (1.02–1.61)	0.033	1.31 (1.05–1.65)	0.019
Moderate	1.25 (1.01–1.55)	0.038	1.28 (1.04–1.58)	0.023
Major	1.89 (1.55–2.31)	<0.001	1.96 (1.61–2.39)	<0.001
Days from hospitalization to nutritional consultation	1.00 (1.00–1.00)	0.678	1.00 (1.00–1.00)	0.843
EN during hospitalization	0.96 (0.86–1.07)	0.488	0.94 (0.84–1.04)	0.230
PN during hospitalization	1.01 (0.91–1.11)	0.860	1.00 (0.91–1.10)	0.993
AN during hospitalization	1.14 (1.01–1.28)	0.030	1.09 (0.97–1.23)	0.137

Note: Model 1 adjusted for age, gender Neurological disease, Lung disease, Kidney disease, Cancer, Infection, Malnutrition, Cardiovascular disease, and Diabetes mellitus; Model 2 adjusted for age, gender and Pluripathology.

**Table 4:** Linear regression model.

	MODEL 1		MODEL 2	
	β (SE)	*p*	β (SE)	*p*
GNRI, ref. No risk				
Low	−5.37 (1.42)	<0.001	−5.69 (1.42)	<0.001
Moderate	−7.29 (1.29)	<0.001	−7.63 (1.29)	<0.001
Major	−9.79 (1.24)	<0.001	−10.29 (1.23)	<0.001
Days from hospitalization to nutritional consultation	0.01 (0.00)	0.083	0.01 (0.00)	0.112
EN during hospitalization	1.88 (0.73)	0.010	1.98 (0.71)	0.005
PN during hospitalization	6.84 (0.64)	<0.001	6.58 (0.64)	<0.001
AN during hospitalization	5.87 (0.70)	<0.001	5.67 (0.70)	<0.001

Note: Model 1 adjusted for age, gender Neurological disease, Lung disease, Kidney disease, Cancer, Infection, Malnutrition, Cardiovascular disease, and Diabetes mellitus; Model 2 adjusted for age, gender and Pluripathology.

## Data Availability

Data available on request from the corresponding author due to privacy reasons. Data are not publicly available due to privacy reasons.

## Supplementary Materials

The following supporting information can be downloaded at: https://www.mdpi.com/article/10.3390/nu17183021/s1, Table S1: Cox proportional hazards model; Table S2: Linear regression model.

## Abbreviations

AN	Artificial Nutrition
BMI	Body Mass Index
CRP	C reactive Protein
EN	Enteral Nutrition
GNRI	Geriatric Nutritional Risk Index
LOS	Length of Hospital Stay
MNT	Medical Nutritional Therapy
MUST	Malnutrition Universal Screening Tool
PN	Parenteral Nutrition
