# Supplementary material for: Undernourished and Undertreated: The Role of Nutritional Care in Geriatric Hospital Outcomes
Source: Nutrients. 2025 Sep 22;17(18):3021. doi: 10.3390/nu17183021 (PMC12472704; doi:10.3390/nu17183021)
Supplement: Supplementary file 1 [file nutrients-17-03021-s001.zip › nutrients-3837127-supplementary.pdf]

Supplementary Materials

Detailed data analyzes Cox proportional hazards model and Linear regression model

Supplementary Table S1. Cox proportional hazards model.

	MODEL 1		MODEL 2	
	**HR(95%CI)**	**p**	**HR(95%CI)**	**p**
GNRI, ref. No risk				
Low	1.28(1.02-1.61)	0.033	1.31(1.05-1.65)	0.019
Moderate	1.25(1.01-1.55)	0.038	1.28(1.04-1.58)	0.023
Major	1.89(1.55-2.31)	<0.001	1.96(1.61-2.39)	<0.001
Male gender, ref. Female	1.17(1.06-1.29)	0.002	1.22(1.11-1.34)	<0.001
Age	1.04(1.03-1.05)	<0.001	1.04(1.03-1.05)	<0.001
Neurological disease	0.99(0.89-1.10)	0.822	-	-
Lung disease	1.23(1.09-1.39)	0.001	-	-
Kidney disease	1.40(1.26-1.56)	<0.001	-	-
Cancer	1.32(1.13-1.55)	<0.001	-	-
Infection	0.84(0.48-1.49)	0.557	-	-
Malnutrition	0.59(0.22-1.58)	0.295	-	-
Cardiovascular disease	1.07(0.97-1.18)	0.179	-	-
Diabetes mellitus	1.11(0.98-1.25)	0.091	-	-
Pluripathology	-	-	1.22(1.08-1.38)	0.001
	**HR(95%CI)**	**p**	**HR(95%CI)**	**p**
Days from hospitalization to nutritional consultation	1.00(1.00-1.00)	0.678	1.00(1.00-1.00)	0.843
Male gender, ref. Female	1.20(1.08-1.32)	<0.001	1.25(1.14-1.38)	<0.001
Age	1.04(1.03-1.05)	<0.001	1.04(1.03-1.05)	<0.001
Neurological disease	1.03(0.93-1.14)	0.569	-	-
Lung disease	1.26(1.11-1.42)	<0.001	-	-
Kidney disease	1.48(1.33-1.65)	<0.001	-	-
Cancer	1.40(1.20-1.64)	<0.001	-	-
Infection	0.94(0.53-1.66)	0.825	-	-
Malnutrition	0.60(0.22-1.60)	0.308	-	-
Cardiovascular disease	1.05(0.95-1.16)	0.336	-	-
Diabetes mellitus	1.10(0.98-1.24)	0.100	-	-
Pluripathology	-	-	1.27(1.12-1.43)	<0.001
	**HR(95%CI)**	**p**	**HR(95%CI)**	**p**
PN during hospitalization	1.01(0.91-1.11)	0.860	1.00(0.91-1.10)	0.993
Male gender, ref. Female	1.19(1.08-1.32)	<0.001	1.25(1.13-1.38)	<0.001
Age	1.04(1.03-1.05)	<0.001	1.04(1.03-1.05)	<0.001
Neurological disease	1.03(0.93-1.14)	0.554	-	-
Lung disease	1.26(1.11-1.42)	<0.001	-	-
Kidney disease	1.48(1.32-1.64)	<0.001	-	-
Cancer	1.41(1.20-1.64)	<0.001	-	-
Infection	0.94(0.53-1.66)	0.824	-	-
Malnutrition	0.60(0.22-1.60)	0.304	-	-
Cardiovascular disease	1.05(0.96-1.16)	0.296	-	-
Diabetes mellitus	1.11(0.98-1.24)	0.095	-	-
Pluripathology	-	-	1.27(1.12-1.43)	<0.001
	**HR(95%CI)**	**p**	**HR(95%CI)**	**p**

AN during hospitalization	1.14(1.01-1.28)	0.030	1.09(0.97-1.23)	0.137
Male gender, ref. Female	1.19(1.08-1.32)	<0.001	1.25(1.13-1.38)	<0.001
Age	1.04(1.03-1.05)	<0.001	1.04(1.03-1.05)	<0.001
Neurological disease	1.02(0.92-1.13)	0.724	-	-
Lung disease	1.27(1.12-1.43)	<0.001	-	-
Kidney disease	1.49(1.33-1.66)	<0.001	-	-
Cancer	1.41(1.20-1.64)	<0.001	-	-
Infection	0.96(0.54-1.70)	0.897	-	-
Malnutrition	0.60(0.22-1.59)	0.302	-	-
Cardiovascular disease	1.06(0.96-1.17)	0.256	-	-
Diabetes mellitus	1.11(0.98-1.25)	0.088	-	-
Pluripathology	-	-	1.27(1.12-1.44)	<0.001

Supplementary Table S2. Linear regression model.

	MODEL 1		MODEL 2	
	**β(SE)**	**p**	**β(SE)**	**p**
GNRI, ref. No Risk				
Low	-5.37(1.42)	<0.001	-5.69(1.42)	<0.001
Moderate	-7.29(1.29)	<0.001	-7.63(1.29)	<0.001
Major	-9.79(1.24)	<0.001	-10.29(1.23)	<0.001
Male gender, ref. Female	0.81(0.67)	0.227	0.38(0.66)	0.563
Age	-0.27(0.05)	<0.001	-0.25(0.05)	<0.001
Neurological disease	-1.21(0.71)	0.089	-	-
Lung disease	-1.72(0.85)	0.042	-	-
Kidney disease	-1.23(0.78)	0.114	-	-
Cancer	-4.85(1.06)	<0.001	-	-
Infection	10.78(4.06)	0.008	-	-
Malnutrition	7.91(5.15)	0.125	-	-
Cardiovascular disease	0.08(0.65)	0.899	-	-
Diabetes mellitus	-0.46(0.78)	0.556	-	-
Pluripathology	-	-	-2.00(0.78)	0.011
Constant	*55.42(5.02)*	*<0.001*	*56.07(5.03)*	*<0.001*
	**β(SE)**	**p**	**β(SE)**	**p**
Days from hospitalization to nutritional consultation	0.01(0.00)	0.083	0.01(0.00)	0.112
Male gender, ref. Female	0.67(0.68)	0.321	0.19(0.67)	0.772
Age	-0.30(0.05)	<0.001	-0.28(0.05)	<0.001
Neurological disease	-2.12(0.71)	0.003	-	-
Lung disease	-2.14(0.85)	0.012	-	-
Kidney disease	-1.82(0.78)	0.020	-	-
Cancer	-5.96(1.06)	<0.001	-	-
Infection	9.40(4.09)	0.022	-	-
Malnutrition	7.28(5.19)	0.161	-	-
Cardiovascular disease	0.15(0.66)	0.815	-	-
Diabetes mellitus	-0.44(0.79)	0.574	-	-
Pluripathology	-	-	-2.49(0.79)	0.002
Constant	*51.40(5.00)*	*<0.001*	*51.89(5.02)*	*<0.001*
	**β(SE)**	**p**	**β(SE)**	**p**
EN during hospitalization	1.88(0.73)	0.010	1.98(0.71)	0.005
Male gender, ref. Female	0.85(0.68)	0.212	0.43(0.67)	0.525
Age	-0.31(0.05)	<0.001	-0.29(0.05)	<0.001

Neurological disease	-2.40(0.72)	0.001	-	-
Lung disease	-2.00(0.85)	0.019	-	-
Kidney disease	-1.73(0.78)	0.027	-	-
Cancer	-5.70(1.06)	<0.001	-	-
Infection	9.62(4.09)	0.019	-	-
Malnutrition	7.60(5.19)	0.143	-	-
Cardiovascular disease	0.18(0.66)	0.780	-	-
Diabetes mellitus	-0.49(0.79)	0.531	-	-
Pluripathology	-	-	-2.51(0.79)	0.001
Constant	*49.24(5.07)*	*<0.001*	*49.69(5.08)*	*<0.001*
	**β(SE)**	**p**	**β(SE)**	**p**
PN during hospitalization	6.84(0.64)	<0.001	6.58(0.64)	<0.001
Male gender, ref. Female	0.26(0.67)	0.693	-0.23(0.66)	0.725
Age	-0.32(0.05)	<0.001	-0.29(0.05)	<0.001
Neurological disease	-1.85(0.70)	0.008	-	-
Lung disease	-2.10(0.84)	0.013	-	-
Kidney disease	-1.39(0.77)	0.072	-	-
Cancer	-6.60(1.05)	<0.001	-	-
Infection	10.07(4.04)	0.013	-	-
Malnutrition	7.14(5.13)	0.164	-	-
Cardiovascular disease	0.27(0.65)	0.675	-	-
Diabetes mellitus	-0.19(0.78)	0.805	-	-
Pluripathology	-	-	-2.28(0.78)	0.003
Constant	*43.05(5.00)*	*<0.001*	*-2.28(0.78)*	*0.003*
	**β(SE)**	**p**	**β(SE)**	**p**
AN during hospitalization	5.87(0.70)	<0.001	5.67(0.70)	<0.001
Male gender, ref. Female	0.76(0.67)	0.257	0.36(0.66)	0.584
Age	-0.32(0.05)	<0.001	-0.30(0.05)	<0.001
Neurological disease	-2.71(0.71)	<0.001	-	-
Lung disease	-1.76(0.85)	0.038	-	-
Kidney disease	-1.41(0.78)	0.070	-	-
Cancer	-5.95(1.05)	<0.001	-	-
Infection	10.12(4.06)	0.013	-	-
Malnutrition	7.71(5.15)	0.135	-	-
Cardiovascular disease	0.37(0.65)	0.569	-	-
Diabetes mellitus	-0.33(0.78)	0.674	-	-
Pluripathology	-	-	-2.38(0.78)	0.002
Constant	*43.17(5.06)*	*<0.001*	*43.51(5.09)*	*<0.001*
